# Nystagmus and Abducens Nerve Palsy as an Early Presentation of Non-alcoholic Wernicke Encephalopathy

**DOI:** 10.7759/cureus.52121

**Published:** 2024-01-11

**Authors:** Siti Hajar Darussalam, Muhammad Mohd Isa, Rafidah Md Saleh, Adzleen Mohmood, Amirah Mohammad Razali

**Affiliations:** 1 Department of Ophthalmology, Hospital Sultan Abdul Aziz Shah, Universiti Putra Malaysia, Serdang, MYS; 2 Department of Ophthalmology, Faculty of Medicine and Health Sciences, Universiti Putra Malaysia, Serdang, MYS

**Keywords:** vitamin b1 deficiency, ophthalmoplegia, nystagmus, thiamine, wernicke encephalopathy

## Abstract

Wernicke encephalopathy (WE) is a rare but life-threatening syndrome that is commonly associated with chronic alcoholism. It has also been found to be associated with malnutrition, prolonged parenteral nutrition, hemodialysis, hyperemesis gravidarum, gastroplasty, and AIDS. It usually presents as a clinical triad of confusion, ophthalmoplegia, and gait ataxia. Nystagmus is usually the most common and earliest ophthalmologic sign. We report a case of non-alcoholic WE in a patient who had prior bariatric surgery and was treated for malnutrition and sepsis, with nystagmus being the initial presentation. The MRI of the brain was normal. The diagnosis of WE was made clinically and was supported by the patient's symptomatic and clinical recovery following intravenous thiamine treatment. It is essential to highlight that a high level of suspicion is needed to diagnose non-alcoholic WE to allow the commencement of appropriate treatment and reduce morbidity and mortality rates related to this condition.

## Introduction

Wernicke encephalopathy (WE) was first identified by Carl Wernicke in 1881 as an "acute superior hemorrhagic polioencephalitis." It was determined in the 1940s that the root cause of WE was found to be thiamine deficiency [1]. During the acute phase of this syndrome, a triad of symptoms manifest, including changes in mental status, ophthalmoplegia, and ataxia [2]. Meanwhile, Korsakoff syndrome (KS) is the chronic phase of WE, characterized by amnesic disorder and confabulations, which was described by Sergei Korsakoff in 1887 [3].

WE typically arise in individuals with alcohol use disorders who have experienced malnourishment due to chronic self-neglect [4]. Wernicke and Korsakoff's various case descriptions implied a diverse etiology for WE. Even though extensive research about WE and KS has been done, they have not identified the primary cause of nutritional deficiency. It was not until five or more decades later that the association between WE and vitamin deficiency was found by Captain de Wardener and Lennox [3]. Thiamine, commonly referred to as vitamin B1, functions as a coenzyme for numerous enzymes in organic pathways, playing a pivotal role in maintaining cerebral energy homeostasis [5].

In contemporary medicine, individuals with significantly reduced intake or those engaging in prolonged fasting can exhibit similarities to the malnourished patients as described by de Wardener and Lennox in the development of WE. Thus, the non-alcoholic WE group includes less prevalent risk factors, such as in patients who received total parenteral nutrition without thiamine replacement [4]. Additional at-risk groups include individuals with chronic gastrointestinal losses, pregnant women experiencing hyperemesis gravidarum, individuals encountering complications after bariatric surgery, and those with inflammatory bowel disease [4].

It is crucial to consider WE in patients displaying any element of the clinical trial, particularly if an identifiable risk factor is present. WE may exhibit diverse ocular manifestations, of which nystagmus is the most prevalent and earliest presentation, with other manifestations including abducent nerve palsy and conjugate gaze palsy [5]. Confirmation of the diagnosis does not rely on a single diagnostic study; instead, it is primarily a clinical diagnosis [6]. A rapid clinical response to intravenous thiamine repletion can help support the correct diagnosis. We report a case of a young gentleman who developed WE after post-complicated bariatric surgery.

## Case presentation

A 38-year-old non-alcoholic, morbidly obese gentleman with a BMI of 42 underwent a laparoscopic sleeve gastrectomy, which involves the removal of the outer margin of the stomach to restrict food intake. He managed to lose 20kg after his bariatric surgery, but unfortunately, he started to have complications one month after that. He complained of a three-day history of vomiting, abdominal distension, and constipation. He was found to have a gastro-cutaneous fistula, gastric perforation, and intraabdominal sepsis, thus requiring a right hemicolectomy and end ileostomy formation. Post-operatively, he was kept nil by mouth and was started on intravenous total parenteral infusion (TPN). Unfortunately, the patient developed methicillin-resistant Staphylococcus aureus sepsis from his infected peripherally inserted central catheter and was started on intravenous vancomycin.

**Figure 1:**
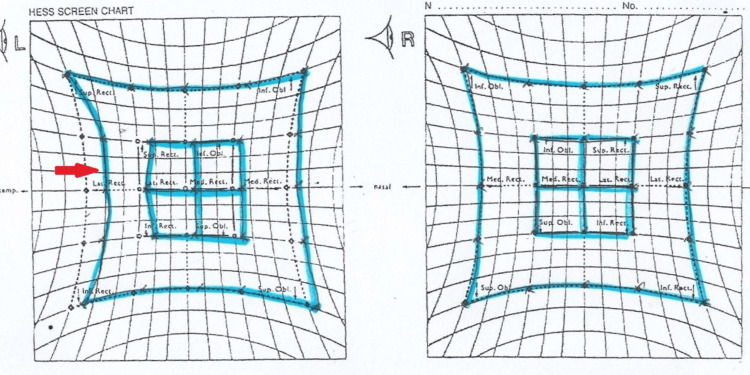
Hess chart examination at presentation showed slightly limited abduction of the left eye (red arrow)

At week 12 of his TPN administration, he complained of oscillopsia and blurring of vision upon lateral gaze only, but no diplopia. He also developed nausea, vomiting, and dizziness. On examination, his Glasgow Coma Scale was 15/15. Visual acuity was 6/6 bilaterally, with no relative afferent pupillary defect. At primary gaze, the patient was orthophoric with no nystagmus present. There was horizontal nystagmus on the lateral gazes. The anterior and posterior segment examinations were unremarkable. Other cranial nerve examinations were normal, including no past pointing and no dysdiadochokinesia. Gait's assessment was difficult as the patient had a gastro-cutaneous fistula and a stoma, which led him to be mainly bedbound and only occasionally mobilized with a walking frame throughout the admission. The Hess chart revealed a limited abduction of 5 degrees over the left eye, as shown in Figure 1. Four days following that, he became drowsy and confused. Neurological examination showed reduced power of the bilateral upper and lower limbs with persistent horizontal nystagmus. An urgent MRI of the brain, however, was normal.

**Figure 2:**
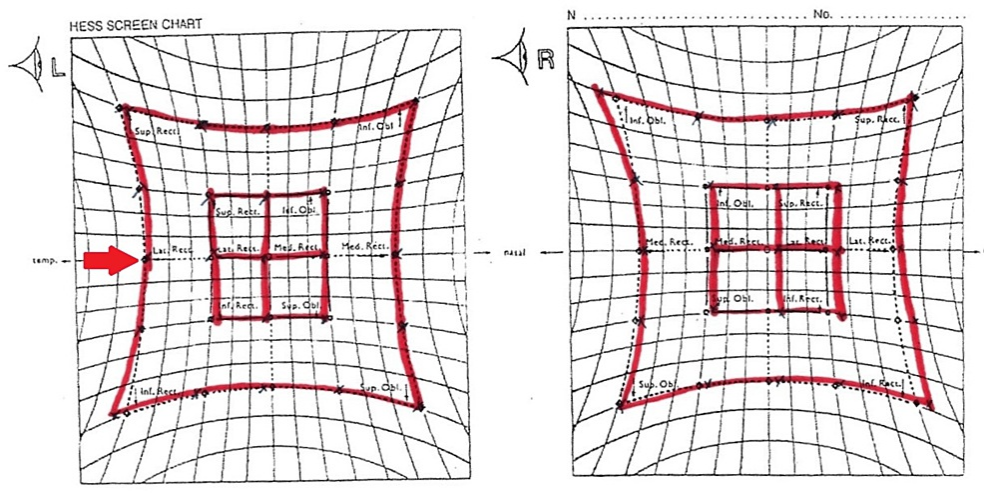
The Hess chart examination at one month showed full recovery of the left eye abduction (red arrow)

His blood investigation revealed he was anemic with a hemoglobin level of 8.3 g/dl and had acute kidney injury with urea of 11.7 g/dL and creatinine of 170 umol/L. Other blood investigations, including magnesium, phosphate, calcium, liver function tests, thyroid function tests, and serum cortisol, were normal. Unfortunately, the thiamine level was not taken as this test was unavailable at our lab. A diagnosis of WE was made based on his clinical presentation by the neurologist, and the patient was treated with intravenous thiamine 500 mg three times daily, which improved his neurologic symptoms within three days. The thiamine dosage was tapered to 250 mg three times daily following the resolution of his neurological symptoms. There was complete resolution of his delirium, with the abducens nerve palsy completely resolved in one month (Figure 2). His horizontal nystagmus decreased but persisted even after six months of follow-up.

## Discussion

The WE constitutes an acute neuropsychiatric emergency arising from a deficiency in thiamine (vitamin B1). Thiamine functions as a crucial coenzyme for diverse metabolic processes in brain cells. It is integral to carbohydrate metabolism for cellular energy production, lipid metabolism to maintain the integrity of the myelin sheath, and amino acid metabolism for the synthesis and proper functioning of neurotransmitters [2]. The body stores of thiamine are only enough for 18 days, and after three weeks, the blood levels also start to fall [2]. Cellular damage starts after four days of thiamine deficiency, and by 14 days, neuronal necrosis may occur, leading to irreversible structural brain lesions [2].

The majority of WE cases are attributed to alcoholics. However, individuals facing malnutrition due to factors such as hyperemesis, starvation, dialysis, cancer, or gastric surgery are also at high risk. When diagnosed in these non-alcoholic individuals, it is referred to as non-alcoholic WE [7]. Patients diagnosed with non-alcoholic WE tend to be relatively young [4]. Two primary warning signs consistently observed in nearly all reported cases are significant weight loss and episodes of vomiting preceding the onset of WE [4]. Bariatric surgery is a procedure that restricts the size of the stomach and gastrointestinal tract, which assists obese patients in achieving a healthier body weight. However, the procedure causes a significant reduction in the absorption of thiamine. In most reported cases of WE following bariatric surgery, patients experience vomiting before the onset of WE [4]. Weight loss typically signals inadequate nutritional intake or the loss of nutrients within the body. In the case of non-alcoholic WE, a challenge may arise from the low level of suspicion due to its atypical presentation.

WE is characterized by the classical triad of symptoms, which includes altered mental status, ophthalmoplegia, and ataxia [2]. The full triad of symptoms in WE is observed in only 10-33% of patients, typically occurring in an advanced thiamine-deficient state [2]. Mental status change is more commonly observed in non-alcoholic WE. Additionally, it has been reported that mental status change is a prominent characteristic in approximately 90% of post-bariatric surgery patients with WE and can vary from apathy to obtundation and, rarely, coma [4-5]. If WE is left untreated, about 80% of cases may progress into KS, which manifests as chronic memory impairment, retrograde or anterograde amnesia, confabulation, and mood instability such as indifference or mild euphoria [8].

With regards to neuro-ophthalmic manifestations of WE, both the afferent and efferent visual systems are commonly affected. Horizontal gaze-evoked nystagmus is the most frequent ophthalmic sign and is considered the earliest sign of thiamine deficiency in WE [5,9]. It progresses into a few stages, with the initial presentation manifesting as a brief, non-sustained nystagmus that further advances into sustained gaze-holding nystagmus without deficit. In the final stage, the nystagmus is accompanied by gaze-holding failure, which indicates the involvement of the nucleus prepositus hypoglossi (horizontal gaze neural integrator) [10]. The next most common ophthalmic finding in WE is bilateral abducens nerve palsy, subsequently conjugate gaze palsies, with horizontal ones more prevalent than vertical ones. Complete ophthalmoplegia is rarely observed in WE, though it is often mentioned as part of the classic triad [11]. Less common ocular manifestations of WE include alterations in the appearance of the optic disc and retina, which include optic disc edema and retinal hemorrhages [10].

It is important to suspect WE in patients displaying any element of the clinical trial, particularly if a risk factor is identified. Even though hematologic studies are available to detect thiamine deficiency, WE is primarily a clinical diagnosis, as there is no critical level below which the patient develops the disorder [6,10,12].

An MRI of the brain is an important tool to help physicians diagnose WE, particularly in cases of non-alcoholic WE, due to its high specificity. It exhibits a high specificity of 93% and a sensitivity of 53% [13]. Only one-half to two-thirds of patients show WE-related MRI findings [5], thus a negative brain MRI cannot definitively rule out the diagnosis of WE. The usual MRI findings include bilateral, symmetric T2-weighted, fluid-attenuated inversion recovery, diffusion-weighted imaging, or T1 post-contrast hyperintensities in the medial thalami, mammillary bodies, periaqueductal area, or tectal plate [13]. Cortical MRI lesions are indicative of permanent damage and serve as a poor prognostic sign. In a study on non-alcoholic Wernicke-Korsakoff syndrome, MRI revealed atrophy in the thalamic area, mammillary bodies, or periaqueductal gray matter, suggesting Wernicke-Korsakoff syndrome in over 66% of cases. In schizophrenia and ulcerative colitis patients, the maximum sensitivity was 100%, whereas in cancer patients, the sensitivity may reach 79% [4]. The lesions found on MRI brain scans can help in supporting the diagnosis of WE; however, they are not pathognomonic.

Hypophosphatemia, Miller-Fisher syndrome, and central pontine myelinolysis are conditions that have similar clinical features as WE and should be considered in the differential diagnosis of WE [14].

WE is a lethal condition, and a delay in treatment may lead to a permanent neurologic deficit or even death. Clinicians should proactively administer thiamine supplementation to patients at risk of developing WE without solely relying on the classic triad of symptoms. In the context of post-bariatric surgery patients, when they lose too much weight rapidly and without adequate consumption, the body’s thiamine stores may become depleted within three to four weeks [4]. In this group of patients, the development of WE can be prevented by providing prophylactic parenteral thiamine treatment [4]. Although the administration of thiamine is acknowledged as crucial, there seems to be a variation in the dose and route of administration. 500 mg of parenteral thiamine three times daily, until symptoms of acute WE resolve, is the recommended protocol as per the European Federation of Neurological Societies and the Royal College of Physicians. This treatment is considered lifesaving and has the potential to reverse this acute neuropsychiatric syndrome [15].

## Conclusions

WE is an acute, life-threatening neurological disorder caused by thiamine deficiency. Since ophthalmoplegia is one of the clinical trails for this condition together with ataxia and confusion, ophthalmologists have a crucial role in recognizing the early symptoms, conducting appropriate assessments, and making the correct diagnosis, especially in high-risk cases such as malnutrition and alcoholics. Early initiation of treatment is necessary as the condition is reversible and, if left untreated, may cause permanent neurological deficits and even death.
